# The Impact of Rainfall on Fecal Coliform Bacteria in Bayou Dorcheat (North Louisiana)

**DOI:** 10.3390/ijerph2006030013

**Published:** 2006-03-31

**Authors:** Dagne D. Hill, William E. Owens, Paul B. Tchounwou

**Affiliations:** 1 Department of Biological Sciences, Grambling State University, P.O. Box 887, Grambling, Louisiana, USA; 2 Louisiana State University Agricultural Experiment Station, Hill Farm Research Station, 11959 Highway 9, Homer, Louisiana, USA; 3 Molecular Toxicology Research Laboratory, NIH-Center for Environmental Health, College of Science, Engineering and Technology, Jackson State University, 1400 Lynch Street, P.O. Box 18540, Jackson, Mississippi, USA

**Keywords:** Non-point source pollution, Fecal coliform, Surface runoff

## Abstract

Fecal coliform bacteria are the most common pollutant in rivers and streams. In Louisiana, it has been reported that 37% of surveyed river miles, 31% of lakes, and 23% of estuarine water had some level of contamination. The objective of this research was to assess the effect of surface runoff amounts and rainfall amount parameters on fecal coliform bacterial densities in Bayou Dorcheat in Louisiana. Bayou Dorcheat has been designated by the Louisiana Department of Environmental Quality as a waterway that has uses such as primary contact recreation, secondary contact recreation, propagation of fish and wildlife, agriculture and as being an outstanding natural resource water. Samples from Bayou Dorcheat were collected monthly and analyzed for the presence of fecal coliforms. Fecal coliforms isolated from these samples were identified to the species level. The analysis of the bacterial levels was performed following standard test protocols as described in Standard Methods for the Examination of Water and Wastewater. Information regarding the rainfall amounts and surface runoff amounts for the selected years was retrieved from the Louisiana Office of State Climatology. It was found that a significant increase in the fecal coliform numbers may be associated with average rainfall amounts. Possible sources of elevated coliform counts could include sewage discharges from municipal treatment plants and septic tanks, storm water overflows, and runoff from pastures and range lands. It can be concluded that non-point source pollution that is carried by surface runoff has a significant effect on bacterial levels in water resources.

## Introduction

It is predicted that by the turn of the next century, water shortages will become more widespread. Predictions from the Water Resource Council allude to increases in the consumption of fresh water by almost 27%. The problem is even worse if groundwater is considered separately from other water sources. The Global 2000 study concluded that pollution, gross national products (GNP), and resource projections all imply rapidly increasing demands for fresh water [1].

According to the U.S. EPA Louisiana 1996 305(b) report, fecal coliform bacteria are the most common pollutant in rivers and streams. It has been noted that of the waterways in Louisiana that were surveyed, 37% of the river miles, 31% of lakes, and 23% of estuarine water in Louisiana had some level of contamination [2]. Possible sources of elevated coliform counts include sewage discharges from municipal treatment plants and septic tanks, storm water overflows, and runoff from pastures and range lands.

Many Louisiana waterways continue to have elevated levels of unhealthy bacteria due to human and natural contaminants. Studies conducted during the summer of 2001 by the Department of Health and Hospitals on the Tangipahoa River showed a strong correlation between high water caused by rain and increased levels of bacteria [3].

Research conducted in 1999 on the Ross Barnett reservoir located in central Mississippi concluded that there exists a potential public health concern with respect to microbial contamination of the water. In most cases in this study, it was shown that the bacterial counts exceeded both the federal and state guidelines for minimizing the health risks associated with water-contact activities [4].

Bursting and overflowing manure lagoons have spawned environmental disasters around the country, sending animal waste flowing into rivers, groundwater and coastal wetlands. In 1995, an 8-acre hog waste lagoon in North Carolina burst, spilling 25 million gallons of animal waste into the New River. The spill killed as many as 10 million fish and closed 364,000 acres of coastal wetlands to shell fishing [5].

According to the water quality standards set by the EPA, *E. coli* is the most reliable of fecal bacterial contamination of surface waters in the U.S. An extensive epidemiological study demonstrated that *E. coli* concentrations are the best predictors of swimming-associated gastrointestinal illness.

The EPA recommended recreational water quality standard for *E. coli* is based on two criteria:

1. A geometric mean of 126 organisms/100ml based on several samples collected during dry weather conditions;
2. 235 organisms/100ml for a single water sample [6]. The geometric mean is calculated by the equation: geometric mean of y = n^th^ root of y_1_ * y_2_ * y_3_…y_n._ If either criterion is exceeded, the site is not in compliance with water quality standards and not recommended for swimming. The current EPA water quality standard for *E. coli* corresponds to approximately 8 gastrointestinal illnesses per 1000 swimmers [7].

This study was designed to analyze the possible impact of monthly rainfall amounts on fecal coliform bacteria levels found in Bayou Dorcheat located in north Louisiana.

## Materials and Methods

### Materials

An API 20E System and an API Profile Recognition System was purchased from BioMerieux, Inc. (Hazelwood, MO).

### Method

A comparison of the fecal coliform level data collected from Bayou Dorcheat for the years of 2002 and 2003 was done. Measurements regarding rainfall levels for the city of Minden, LA located within a one mile radius of Bayou Dorcheat located in north Louisiana were retrieved from the Louisiana Office of State Climatology [8]. According to the Department of Environmental Quality, Bayou Dorcheat has been designated by DEQ as having uses such as primary contact recreation, secondary contact recreation, propagation of fish and wildlife, agriculture and as being outstanding natural resource water [9]. All test conducted were referenced from the 20^th^ edition of the Standard Methods for the Examination of Water and Wastewater [10].

### Collection of Samples

All water samples were collected with a volume of not less than 100ml. A space of at least 2.5 cm was left in the bottle to facilitate mixing by shaking. The containers used were in accordance to the 20^th^ edition of Standard Methods for the Examination of Water and Wastewater. Samples were collected in non-reactive glass or plastic bottles that had been cleansed and rinsed carefully, given a final rinse with distilled water, and sterilized [10]. Containers were lowered to a depth of not greater than 2 ft below the surface to fill. The samples were placed immediately on ice in order to have a temperature of less than 10 °C during a maximum transport time of 6 h [10]. Five duplicate samples from each site were collected once monthly for a period of twelve months. Bayou Dorcheat samples were collected at a distance within two feet from the bank and at a depth of two feet. Samples were collected at least one mile away from community housing to avoid the possibility of septic systems in the area. The Bayou Dorcheat area has public sewage. The area of sample collection used for Bayou Dorcheat had a minimal flow rate.

### Fecal Coliform Count

Ten ml of water sample were vortexed and filtered onto a membrane filter using a sterile filtration unit. The approved technique used was from Clesceri et al. [10].

After filtration, forceps were used to place the membrane filter on an MFC agar plate. The plate was then incubated in an incubator at a temperature of 45°C for 24 hours. The plates were then checked for bacteria colony growth.

### API 20 E. Systems

The API 20E System was used in conjunction with the API Profile Recognition System (bioMerieux, Inc., Hazelwood, MO) so that members of the family *Enterobacteriaceae* and other Gram-negative bacteria could be accurately identified.

The API 20 E strip consists of 20 micro-tubes containing dehydrated substrates. These tests were inoculated with the bacterial sample suspension. Each sample was incubated for 18–24 hrs. at 35–37°C. This system is a standardized, miniaturized version of conventional procedures for the identification of *Enterobacteriaceae* and other Gram-negative bacteria.

### Statistical Analysis

Descriptive statistics were applied to determine the mean values of all rainfall and bacterial parameters evaluated. Standard deviations were computed as measures of variance. Statistical analysis was performed using GraphPad InStat program version 3.00 for Windows 95, GraphPad Software, San Diego, California. The Dunn’s Multiple Comparisons test was applied to determine significant differences in mean values of each studied parameter among the sampling sites. The level of significance was considered at p ≤ 0.05.

## Results

**Figure 1:**
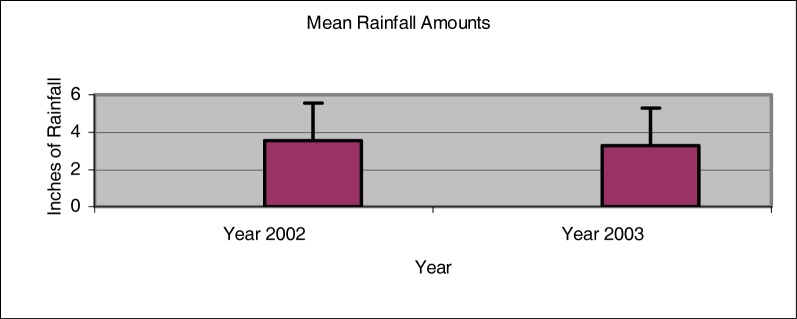
Mean values of rainfall amounts for the years 2002 and 2003 (LOSC 2005).

**Figure 2:**
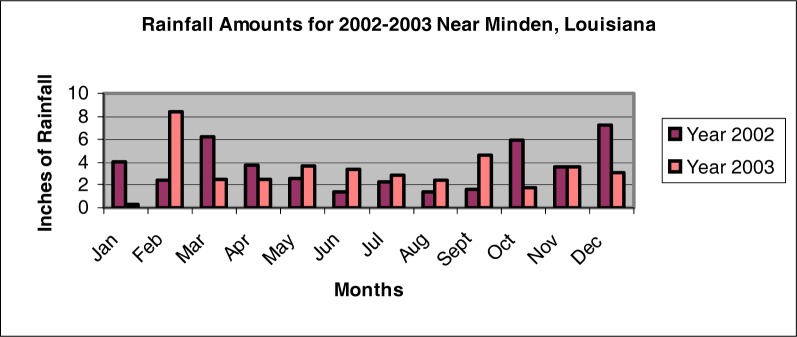
Rainfall amounts for 2002 and 2003 near Minden, Louisiana (LOSC 2005).

The mean monthly rainfall level for the year 2002 was found to be 3.54 ±2.00 inches (Figure 1). The year of 2003 had a slightly lower mean monthly rainfall level of 3.29 ± 1.94 inches. As shown in Figure 2, the greatest monthly rainfall amount during the years 2002 and 2003 occurred during the months of December and February, respectively. Differences found between the mean monthly rainfall levels were determined to be not significant (p > 0.05). The water flow rate for each of the sites was estimated at being minimal.

**Figure 3:**
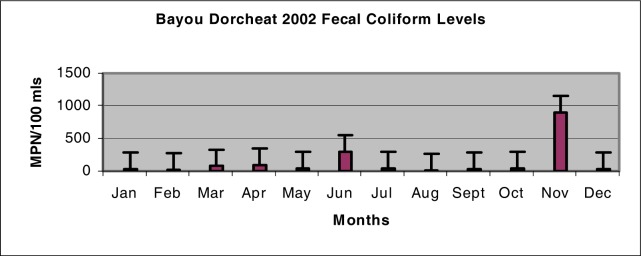
Bayou Dorcheat Fecal Coliform Levels (MPN/100 mls) for 2002.

**Figure 4:**
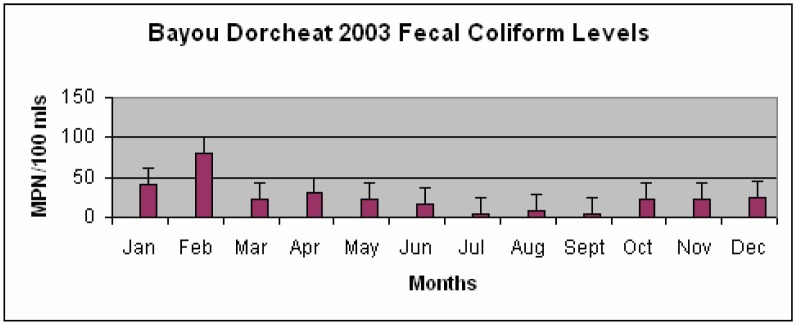
Bayou Dorcheat Fecal Coliform Levels (MPN/100 mls) for 2003

The mean fecal coliform level for the year 2002 was found to be 136.42 ± 252.58 MPN/100 ml while the year 2003 had a much lower mean of 25.17 ± 20.26 MPN/100 ml. Figure 3 shows that the highest fecal coliform levels were detected during the month of November for the year 2002, while according to Figure 4 February had the highest fecal coliform levels detected during the year 2003.

The lowest levels of fecal coliform bacteria for the years 2002 and 2003 were found to be during the months of August and September, respectively. There were no significant differences noted in the fecal coliform levels (p > 0.05) between the years 2002 and 2003. Differences found between the mean monthly rainfall levels for the years 2002 and 2003 to the fecal coliform levels detected during these same years were determined to be not significant (p > 0.05).

## Discussion

According to the Department of Environmental Quality, Bayou Dorcheat has been designated by DEQ as having uses such as primary contact recreation, secondary contact recreation, propagation of fish and wildlife, agriculture and as being outstanding natural resource water [9].

According to the Environmental Protection Agency, Bayou Dorcheat has been included on the list of impaired waters. The suspected cause of this impairment is because of its dissolved oxygen levels [11]. It is possible that the levels of bacteria found within Bayou Dorcheat could have some negative effect on the dissolved oxygen levels.

Although the mean monthly rainfall levels for both years within the study were very similar, the fecal coliform levels were not. The mean bacterial level for the year 2002 was five times higher than the bacterial level for the year 2003. Unknown factors such as increased recreational fishing during this time could possibly account for elevated levels. For surface water used as a primary contact recreation, the fecal coliform content shall not exceed a log mean of 200 CFUs/100 ml [8]. While none of the observed months during the year 2003 exceeded the acceptable criteria level established by DEQ, the year of 2002 had two months in which the level was exceeded.

## Conclusions

It was proven by these observations that the fecal coliform levels within Bayou Dorcheat are not significantly linked to the rainfall level. It can therefore be concluded from the finding of this research that although non-point source pollution has a significant effect on bacterial levels in runoff water and in water resources, this effect would be the result factors other than just the mean monthly rainfall level and not this alone.
